# Evaluation of Positive Cases for Dense Fine Speckled (DFS) Immunofluorescence Pattern and Anti-DFS70 Antibodies

**DOI:** 10.12669/pjms.41.2.10336

**Published:** 2025-02

**Authors:** Melahat Gurbuz, Betul F. Yıldırım, Yeliz Cetinkol

**Affiliations:** 1 Melahat Gurbuz, MD Assistant Professor, Department of Medical Microbiology, Faculty of Medicine, Afyonkarahisar Health Sciences University, Afyonkarahisar, Turkey; 2 Betul F. Yıldırım, MD Department of Medical Microbiology, Faculty of Medicine, Afyonkarahisar Health Sciences University, Afyonkarahisar, Turkey; 3 Yeliz Cetinkol, MD Professor, Department of Medical Microbiology, Faculty of Medicine, Afyonkarahisar Health Sciences University, Afyonkarahisar, Turkey

**Keywords:** DFS70, Anti-nuclear antibody, IIF-ANA, immunoblot

## Abstract

**Objective:**

The detection of anti-nuclear antibodies (ANA) is crucial in diagnosing systemic autoimmune rheumatic diseases (SARDs). The Dense Fine Speckled (DFS) nuclear pattern is one of the most common indirect immunofluorescence (IIF) patterns detected during routine ANA screening in patients with various clinical conditions. The aim of this study was to analyze the data of patients who were positive for DFS/antiDFS in our patient population and to show the possible clinical relationship.

**Methods:**

In this retrospective study, 7406 patient serum samples sent to our laboratory for routine ANA screening between May 2022-2023 were evaluated for the presence of anti-DFS.

**Results:**

In a group of patients referred for routine ANA screening using the indirect immunofluorescence method, the frequency of DFS pattern was found to be 4.55% (337/7406), with ANA positivity detected at a rate of 25.68% (1902/7406). Out of 221 patients with DFS pattern, 181 tested positive for anti-DFS antibodies in both the IIF-ANA and immunoblot (IB) tests. Additionally, 11 of these patients tested positive for other antibodies against different extractable nuclear antigens (anti-ENAs). Conversely, only seven out of the 40 patients who tested negative for anti-DFS antibodies showed positive results for other anti-ENAs.

**Conclusion:**

The DFS pattern is often positive in individuals. In patients exhibiting this pattern, anti-DFS70 antibody may be detected alone or in combination with SARD-associated autoantibodies. Therefore, we propose that in this patient cohort, it would be more prudent to screen for additional concomitant autoantibodies with anti-ENA rather than confirming isolated anti-DFS.

## INTRODUCTION

Demonstrating the presence of antinuclear antibodies (ANA) is a crucial diagnostic marker for systemic autoimmune rheumatic diseases (SARDs), including systemic lupus erythematosus (SLE), systemic sclerosis, Sjogren’s syndrome, dermatomyositis/polymyositis (DM/PM), and mixed connective tissue disease.^1^,^2^ ANA can be detected in a variety of conditions, including thyroid disease, infection, malignancy, and even in healthy individuals.^3^

The proposed “gold standard” method for ANA screening is indirect immunofluorescence (IIF) using a human epithelial (HEp-2) cell substrate.^2^,^4^ The dense fine speckled (DFS) nuclear pattern is frequently observed in ANA screening tests, often at very high titers.^2^ The DFS pattern is identified as an intense and heterogeneous punctate staining of the nucleus of interphase cells and the plate of metaphase cells and classified as AC-02 pattern by the International Consensus on ANA Patterns (ICAP) committee.^5^,^6^

The presence of autoantibodies (anti-DFS70) against a 70 kDa protein called ‘DNA-binding transcription coactivator of transcription p75’ or ‘lens epithelium-derived growth factor’ (LEDGF) has been associated with this pattern.^7^ It was first described in 1994 and has since been detected in many patients without autoimmune disease and even in healthy individuals.^8^ DFS70/LEDGFp75 is a versatile protein with multiple roles in stress response and transcriptional regulation. It has been implicated in various physiological and pathological conditions, including autoimmunity, cancer, acquired immunodeficiency syndrome (AIDS), and ocular diseases.^8^,^9^

The IB method, commonly used for the detection of specific autoantibodies against extractable nuclear antigens (ENAs) associated with certain SARDs, is usually applied after a positive ANA-IIF test assisting the clinician in the classification of the patient and the confirmation of the presence of specific autoantibodies. Additionally, the ability to detect multiple autoantibodies in a single study is beneficial.^10^,^11^

The clinical significance of DFS pattern/anti-DFS70 antibodies has not been fully clarified. The fact that they are detected more frequently in healthy individuals than in those with SARDs suggests that isolated anti-DFS70 antibodies may be considered a reliable marker to exclude SARD in the absence of SARD-associated autoantibodies.^12^,^13^ However, some studies indicate that supporting this suggestion is challenging due to the lack of difference in the prevalence of anti-DFS70 positive samples between SARD and non-SARD groups.^14^ The objective of this study was to determine the regional prevalence of anti-DFS on samples sent for routine ANA screening and to analyze DFS positivities to investigate any potential clinical relationships.

## METHODS

This study retrospectively analyzed 7406 patient samples sent to the Medical Microbiology Laboratory of Afyonkarahisar Health Sciences University Health Application Research Center (AFSU-SUAM), a tertiary hospital, for routine ANA screening between May 2022 and May 2023.

### Ethical Approval:

The study was conducted with the approval of the Clinical Research Ethics Committee at Afyonkarahisar Health Sciences University (Ref.: 2023/10).

### ANA IIF Test Method:

ANA screening was performed using the HEp 20-10 kit (Euroimmun AG, Luebeck, Germany) with the IIF-ANA method at an initial dilution of 1/100, as recommended by the manufacturer. The ANA IIF patterns were classified according to ICAP standards (www.ANApatterns.org).

### Immunoblot Test Method:

The EUROLINE ANA Profile et Mi-2, Ku, 3 plus DFS70 (IgG) test kit (Euroimmun AG, Germany) was used to investigate antibodies against extractable nuclear antigens (ENA) and DFS70 through the IB method. The testing procedure was performed using the EUROBlotOne system (Euroimmun) according to the manufacturer’s recommendations. The strips were then automatically dried and photographed. Band intensities were evaluated using EUROLineScan software (Euroimmun).

### Statistical analysis:

The arithmetic mean and standard deviation values for quantitative data were calculated, along with the number and percentage of qualitative data, using the MS Office Excel and IBM SPSS 26.0 (Statistical Package for Social Sciences) software.

## RESULTS

Results of 7406 patients (mean age ± SD= 45.14 ± 17.54 years) referred from different clinics for routine ANA testing were analyzed. ANA positivity (≥1/100) was detected in 25.68% (1902/7406) of the patients, with a frequency of DFS pattern among all patients at 4.55% (337/7406) according to the IIF-ANA test. Notably, the proportion of female patients was high in both ANA positive (80.8%) and DFS pattern positive (81.6%) groups (Table-I).

**Table-I:** Characteristics of the patient group.

Characteristics	ANA test order n (%)	ANA positive n (%)	DFS pattern positive n (%)	Anti-DFS antibody positive n (%)
Number	7406 (100)	1902 (25,68)	337/1902 (17,71)	181/221 (81,9)
Mean age ± SD	45,14 ± 17,5	46,38 ± 17,4	40,52 ± 17,4	41,55 ± 14,8
***Gender***				
Female	5266 (71,1)	1537 (80,8)	275 (81,6)	158 (87,3)
Male	2140 (28,9)	365 (19,2)	62 (18,4)	23 (12,7)
Female/Male	2,4	4,2	4,4	6,8

Among patients with a positive DFS pattern, 91.4% (308 patients) did not have a diagnosis of SARD. Musculoskeletal complaints were the most common presentation among patients without a SARD, accounting for 61.7% (190 patients). Rheumatoid arthritis was the most common diagnosis among the patients with a positive DFS pattern and SARD diagnosis, observed in 19 out of 29 patients (65.5%) (Table-II).

**Table-II:** Characteristics of DFS pattern positive patients.

Patients without SARD diagnosis	n	%	Patients with SARD diagnosis	n	%
Mean ± SD= 40,2 ± 17,5	308	91,4	Mean ± SD= 40,4 ± 16,7	29	8,6
Female	249	80,8	Female	26	89,7
Male	59	19,2	Male	3	10,3
Disease			Disease		
Musculoskeletal complaints	190	61,7	RA	19	65,5
General examination	60	19,5	Sjögren’s syndrome	4	13,8
Gastrointestinal system diseases	27	8,8	Mixed connective tissue disease	3	10,3
Dermatological diseases	19	6,2	SLE	2	6,9
Neurological diseases	10	3,2	Scleroderma	1	3,5
Hematological diseases	2	0,6			

SARD: Systemic Autoimmune Rheumatic Disease, RA: Rheumatoid arthritis, SLE: Systemic Lupus Erythematosus.

DFS pattern positivity was mostly observed in rheumatology patients 59.6% (201 patients). The Internal Medicine clinic and the Physical Therapy and Rehabilitation (PTR) clinics accounted for 11.3% (38 patients) and 10.4% (35 patients) of the patient samples, respectively. Anti-DFS positivity was detected in 66.9% of samples from rheumatology, 16% of samples from PTR, and 7.7% of samples from Internal Medicine. Anti-DFS70 antibody was confirmed in 81.9% of the 221 samples where DFS pattern was detected in IIF-ANA screening and IB test was performed. Among the 181 anti-DFS70 positive patients, 6.1% showed an association with SARD-related anti-ENA. The frequently associated antibodies were Mi-2, Ro52, PM-Scl, and RNP/Sm. One patient had anti-DFS70, PM-Scl, and dsDNA, while another patient had anti-DFS70, PM-Scl, and Ku. After a thorough analysis of the complaints of these 11 patients, the most common complaint was joint pain in 3 (27.3%) patients. Among the 40 patient samples that tested negative for anti-DFS, 7 (17.5%) were found to have SARD-associated anti-ENA, with PM-Scl being the most commonly detected antibody (Table-III).

**Table-III:** Other autoantibodies detected by immunoblot and clinical findings in DFS pattern-positive patients.

DFS pattern/ Anti-DFS70	Number	Autoantibodies (Complaint/diagnosis)
+/+	181	1 PM-Scl, dsDNA (RA)
		1 PM-Scl, Ku (pain)
		1 Mi-2 (AS)
		1 Mi-2 (joint pain)
		1 Ro-52 (DM, joint pain)
		1 Ro-52 (baş ağrısı)
		1 RNP/Sm (AS)
		1 RNP/Sm (AS)
		1 dsDNA (SLE)
		1 Scl-70 (joint pain)
		1 AMA-M2 (PBS)
+/-+/-+/-	40	2 PM-Scl (connective tissue systemic involvement)
		1 PM-Scl (pain)
		1 PM-Scl (DM)
		1 Scl-70 (Morphea)
		1 SS-B (joint pain)
		1 SS-A (SLE)

DFS: Dense fine speckled, PM-Scl: Polimiyozit-skleroderma, dsDNA: double stranded DNA, RA: Rheumatoid arthritis, AS: Ankylosing spondylitis, DM: Diabetes mellitus, SLE: Systemic Lupus Erythematosus, RNP/Sm: Ribonucleoprotein / Smith antigen, Scl-70: DNA topoisomerase I, AMA-M2: Mitochondria antigen M2, PBC: Primary biliary cirrhosis.

## DISCUSSION

This study demonstrated the frequency of the IIF-DFS pattern and anti-DFS70 antibodies in ANA-positive individuals, the prevalence in the patient population we serve, and the potential clinical relevance by analyzing the records of patients with DFS. The intensely stained metaphase chromosome plate is an important feature of DFS pattern also known as the AC-2. Autoantibodies that produce this pattern target the DFS protein, specifically DFS70. In some studies, AC-2 patterns that do not show anti-DFS70 reactivity have been defined as ‘pseudo-DFS’ patterns.^6^,^9^

Our study demonstrated that 25.68% (1902/7406) of patients referred for routine ANA testing had ANA positivity (≥1/100), which is consistent with the literature from Turkey, showing similar rates 23.7% and 20.6%.^3^,^15^ On the contrary, in a Korean study of 5509 patients, ANA positivity was 11.6%^16^, while in two large-scale studies in China it was 32% and 34.9%.^17^

The frequency of DFS pattern was 4.55% (337/7406). The mean age of the patients was 40 years and 81.6% were female. Studies conducted in our country found this rate to be 6%3, 5.1%^18^, and 5%^15^, respectively. When examining studies conducted abroad, this rate was found to be 4.3% in Brazil^19^, 2.3% in Korea^16^, 6.4% in Singapore^20^, 1.27% in China^14^, and 1.14% in China.^17^ Variations in positivity rates may be due to heterogeneity in the composition of the ANA screening groups studied, such as gender, age, ethnicity, and reasons for requesting ANA tests, as well as differences in the screening method.

When patients with a positive DFS pattern were classified according to their diagnosis, it was found that 308 (91.4%) of these patients did not have a diagnosis of SARD. Upon analyzing the reasons for presentation to the clinics of patients without a SARD diagnosis, musculoskeletal complaints were the most common, observed in 190 (61.7%) patients. In a study it was found that 89.2% of patients with DFS70 IIF staining pattern had non-SARDs, with most common complaint as musculoskeletal issues (47.4%).^3^ In Zhang et al.’s study, 81.8% of patients had non-SARDs, with infective diseases being the most frequently observed.^14^

Among the 29 (8.6%) patients with a positive DFS pattern and SARD diagnosis, rheumatoid arthritis was observed in 19 (65.5%) patients. Studies have reported SARD diagnosis in 10.8% and 18.2% of patients with DFS70 IIF staining pattern, respectively. Similar to our study, the most common diagnosis of SARD in both studies was RA.^3^,^14^

Similar to this study, a study conducted in China found that the most common IIF-DFS pattern positivity was found in samples from rheumatology clinics.^14^ In the analysis of anti-DFS positivity, 121 (66.9%) samples from rheumatology were positive, followed by 29 (16%) samples from PTR and 14 (7.7%) samples from Internal Medicine.

The presence of DFS was confirmed by IB in 181 (81.9%) of 221 samples where the DFS pattern was detected in the IIF-ANA test and an IB test was requested. This rate is similar with a study conducted in Turkey (82.1%), but higher than others (75.3% and 41%, respectively).^2^,^5^ In our study, we found an association between SARD-related anti-ENA in 11 (6.1%) of 181 anti-DFS70 positive patients. The most frequently associated antibodies were Mi-2, Ro52, PM-Scl, and RNP/Sm. Analyzing the complaints of these 11 patients, joint pain was the most common (27.3%). In different studies, SARD-related anti-ENA positivity in patients with anti-DFS70 positivity was found to be 5.9%^12^, 7.1%^3^, 7.2%^5^ and 26.9%^2^, respectively with Ro52 being the most frequently detected. SARD-related anti-ENA was detected in 7 (17.5%) out of 40 patient samples that tested negative for anti-DFS. The most commonly detected antibody was PM-Scl (Table-III).

The most common reason for ANA testing is to aid in the diagnosis of SARDs. The DFS pattern was found to be frequently positive in various diseases and in healthy individuals.^7^ The clinical significance of the DFS pattern is still unknown.^14^ Some studies suggest that DFS pattern/anti-DFS70 positivity may serve as an exclusion criterion for SARDs.^11^,^16^,^21^-^23^ While some point out that DFS pattern/anti-DFS70 positivity or ENA autoantibody negativity as exclusion criteria for SARDs may result in missed diagnoses. As its chronic and slow course nature longer follow-up studies are necessary.^17^

Studies have reported that the presence of DFS pattern in IIF-ANA scan does not necessarily indicate the presence of anti-DFS70 antibodies. Additionally, the DFS pattern may be accompanied by SARD-associated autoantibodies. Therefore, it is important to conduct specific tests for SARD-related autoantibodies in addition to testing for anti-DFS70 when a DFS pattern is observed.^3^,^5^

In this study, we conducted a retrospective analysis of patient results from ANA screening. The majority of patients (81.9%) with a DFS pattern were confirmed by the IB method, and ENA profiles were also determined. The test was used to investigate comorbidities in patients with anti-DFS70 antibodies and to explore the clinical significance of this antibody.

### Limitations:

Since it was a retrospective study, 40 patients with DFS pattern detected in IIF-ANA test could not be validated because they did not have an IB request.

## CONCLUSION

The study results indicate that in patients with DFS pattern, anti-DFS70 antibody can be detected alone or in combination with SARD-related autoantibodies. Therefore, it is not possible to exclude SARDs in the presence of DFS pattern/anti-DFS. Therefore, we recommend investigating SARD-associated autoantibodies together instead of solely confirming anti-DFS70 using an additional method for patients with DFS pattern detected in ANA screening.

### Author Contribution:

**MG:** Conceptualization, Data curation, Formal Analysis, Methodology, Writing - original draft, Writing - review & editing.

**BFY:** Conceptualization, Data curation, Supervision, Critical review, Writing - original draft, Writing - review & editing.

**YC:** Conceptualization, Methodology, Writing - original draft, Writing - review & editing.

All authors have approved the final version and are accountable for the integrity of the study.
